# Salivary malondialdehyde as an oxidative stress biomarker in oral and systemic diseases

**DOI:** 10.15171/joddd.2016.011

**Published:** 2016-06-15

**Authors:** Maryam Khoubnasabjafari, Khalil Ansarin, Abolghasem Jouyban

**Affiliations:** ^1^Assistant Professor, Tuberculosis and Lung Disease Research Center, Tabriz University of Medical Sciences, Tabriz 51664, Iran; ^2^Professor, Tuberculosis and Lung Disease Research Center, Tabriz University of Medical Sciences, Tabriz 51664, Iran; ^3^Professor, Pharmaceutical Analysis Research Center and Faculty of Pharmacy, Tabriz University of Medical Sciences, Tabriz 51664, Iran

Oxidative stress is the imbalance between oxidative status and the antioxidant levels in the biological system. A number of biomarkers are routinely used in clinical investigations to measure this imbalance, including malondialdehyde (MDA), F_2_-isoprostanes, vitamins A, C and E, carotenes, retinol, lipid hydroperoxides, protein carbonyl, total thiol, total antioxidant capacity, etc.

Saliva is a more attractive biological sample for clinical studies on oral diseases. In a recent review article,^1^ the advantages of saliva as an alternative biological sample for diagnosis, prognosis and therapeutic responsiveness of some diseases were discussed. Variations in the salivary concentrations of a number of biomarkers of oxidative stress were reviewed along with some characteristics of an ideal biomarker. Wang et al^1^ correctly emphasized the low reproducibility of the analytical methods used for quantification of oxidative stress biomarkers in saliva and guidelines were provided for a qualified practice on saliva collection, processing, storage and analysis. Two other review papers were also discussed on saliva analysis in some diseases.^2,3^

The aim of this editorial is to provide further support for variations in one of the reviewed biomarkers, i.e. MDA. There are a number of confounding factors affecting the salivary concentrations of analytes some of which were mentioned in the published work.^1^ To show the very wide variations in MDA concentrations in saliva among various research groups, the salivary MDA concentrations of healthy control groups in the available reports^4-25^ are listed in the Table. MDA values were measured after derivation with thiobarbitoric acid using the mentioned analytical methods in the last column of the Table.

**Table 1:** Salivary MDA concentrations in the case and control groups of available reports, the number of observations (N), the analytical method used (after derivation with thiobarbitoric acid) and their references

**Disease**	**MDA (nmol/L) of case (N)**	**MDA (nmol/L) of control (N)**	Analytical method*	**Reference**
**Chronic periodontitis**	100 (36)	60 (28)	HPLC	4
**Chronic periodontitis, after therapy**	90 ± 10 (48)	110 ± 30 (35)	HPLC	5
**Chronic periodontitis, before therapy**	110 ± 50 (48)	100 ± 20 (35)	HPLC	5
**Chronic periodontitis, diabetic**	10790 ± 8070 (30)	1530 ± 1300 (30)	UV 532 nm	6
**Chronic periodontitis, non-diabetic**	9090 ± 8160 (30)	1530 ± 1300 (30)	UV 532 nm	6
**Chronic periodontitis (men)**	~ 4.2 mmol/g protein (9)	1.5 mmol/g protein (11)	F	7
**Chronic periodontitis (women)**	~ 3 mmol/g protein (14)	1.5 mmol/g protein (8)	F	7
**Crohn’s disease**	~ 1150 ± 200 (16)	~ 900 ± 150 (16)	UV 532 nm	8
**Crohn’s disease**	146 ± 64 (28)	27 ± 19 (20)	F	9
**Diabetes**	650 ± 130 (25)	230 ± 70 (25)	UV 335 nm	10
**Diabetes mellitus**	~ 7000 ± 200 (19)	~ 6800 ± 180 (19)	UV 532 nm	11
**Diabetic without chronic periodontitis**	1910 ± 1720 (30)	1530 ± 1300 (30)	UV 532 nm	6
**Down syndrome**	6720 ± 4220 (30)	3960 ± 3650 (30)	UV 530 nm	12
**Fixed orthodontic appliances (posttreatment, 1 month)**	3870 ± 3060 (50)**	-	Caymen kit	13
**Fixed orthodontic appliances (posttreatment, 6 month)**	3600 ± 2450 (50)**	-	Caymen kit	13
**Fixed orthodontic appliances (pretreatment)**	3760 ± 2180 (50)**	-	Caymen kit	13
**Healthy, quid chewing/smoking habit**	217.6 ± 34.1 (30)	181.2 ± 34.1 (35)	UV 532 nm	14
**Oral leukoplakia**	330 ± 70 (40)	80 ± 70 (40)	UV 535	15
**Oral leukoplakia**	651 ± 80 (20)	349 ± 90 (20)	UV 532 nm	16
**Oral leukoplakia**	417.5 ± 32.1 (50)	181.2 ± 34.1 (35)	UV 532 nm	14
**Oral lichen planus**	430 ± 7 (40)	80 ± 70 (40)	UV 535	15
**Oral lichen planus**	2030 ± 810 (21)	1470 ± 370 (20)	UV 535	17
**Oral lichen planus**	~ 3.5 ± 0.1 (32)	3.2 ± 0.1 (30)	UV 532 nm	18
**Oral lichen planus**	~ 5800 ± 2000 (36)	~ 3200 ± 1600 (36)	UV 532 nm	19
**Oral premalignant lesions**	~ 580 ± 420 (16)	~ 220 ± 160 (16)	UV 532 nm	20
**Oral squamous cell carcinoma**	1000 ± 210 (40)	80 ± 70 (40)	UV 535	15
**Oral squamous cell**	~ 3.9 ± 0.3 (26)	~ 3.2 ± 0.1 (30)	UV 532 nm	18
**Oral squamous cell carcinoma**	1007 ± 160 (20)	349 ± 90 (20)	UV 532 nm	16
**Oral squamous cell carcinoma**	930.6 ± 31.9 (50)	181.2 ± 34.1 (35)	UV 532 nm	14
**Oral submucous fibrosis**	430 ± 70 (40)	80 ± 70 (40)	UV 535	15
**Oral submucous fibrosis**	434.4 ± 42.1 (65)	181.2 ± 34.1 (35)	UV 532 nm	14
**Patients received ivBPs without BRONJ**	390 ± 110 (20)	210 ± 90 (17)	UV 532 nm	21
Patients with BRONJ*** received ivBPs****	510 ± 130 (24)	210 ± 90 (17)	UV 532 nm	21
**Periodontitis (posttreatment, non-smokers)**	60	65	F	22
**Periodontitis (posttreatment, smokers)**	60	85	F	22
**Periodontitis (pretreatment, non-smokers)**	95	65	F	22
**Periodontitis (pretreatment, smokers)**	123	85	F	22
**Recurrent aphthous**	526 ± 92 (20)	232 ± 61 (20)	UV 532 nm	23
**Recurrent aphthous**	480 ± 160 (30)	280 ± 120 (20)	HPLC	24
**Smokers (passive)**	4360 ± 680 (20)	3470 ± 650 (20)	UV 532 nm	25
**Smokers, 20 cigarettes/day**	6070 ± 2330 (20)	3470 ± 650 (20)	UV 532 nm	25
**Ulcerative colitis**	~ 1000 ± 100 (16)	~ 900 ± 150 (16)	UV 532 nm	8

^*^ HPLC: High performance liquid chromatography, UV: Ultra-violet, F: Fluorescence. ^**^ We assumed that the MDA values are expressed as mmol/L in the original reference.^13^ ^***^ BRONJ: bisphosphonate-related osteonecrosis of the jaw. ^****^ ivBPs: interavenous bisphosphonates.

As clearly shown in the review article,^1^ controversial findings were reported for most clinical cases. As an example, the salivary MDA values for oral lichen planus were reported 3.5 nmol/L,^18^ 430 nmol/L,^15^ 2030 nmol/L^17^ and 5800 nmol/L.^19^ The corresponding values for the control groups were 3.2, 80, 1470 and 3200 nmol/L, respectively. The data were scattered even for a given research group; as an example the MDA values of the control groups varied from 27^9^ to 680^11^ to 900^8^ nmol/L. These discrepancies were also observed when a single analytical method with the same analytical conditions was used to measure the MDA levels in biological samples.^26^

Careful examination of MDA values in the control groups of the reported results in the Table reveals that they varied from 3.2 nmol/L to 3960 nmol/L (1237 folds), which is an unacceptable variation for healthy controls. Wide variations were also observed for plasma MDA concentrations.^27^ These wide variations might have originated from different sources, including saliva sample collection procedure, storage of samples prior to analysis, and the analytical method. As an example, co-existence of some biochemical agents in saliva could interfere with spectroscopic analysis of MDA and sialic acid is a classical analyte interfering with MDA in biological samples.^28^

Lipid peroxidation, reaction of deoxyribose with a hydroxyl radical, γ–irradiation of carbohydrates and prostaglandin synthesis pathway are the main sources of systemic MDA concentrations. Salivary MDA originates from systemic sources and also its production in the oral cavity. It is also formed in foods and MDA levels in biological samples are affected by smoking and some drugs.^29^^and references therein^ The chemical stability of MDA solutions, its reactions with biochemical agents and metabolism of MDA in biological samples are the other effective parameters. The MDA measurement methods are based on thiobarbitoric acid derivation possess poor reproducibility, low repeatability and non-specificity. More details on the validity of MDA measurements in biological samples were discussed in a recent review article.^29^ These limitations on MDA analysis and its action as a biomarker of oxidative stress have been noticed in a number of publications;^30-37^ however, they have been ignored by some research groups as clearly mentioned.^38^ Interestingly, most clinical studies on MDA variations in pathological conditions published in recent years have used simple spectroscopic analysis whereas the validity of this analytical method is seriously questionable. We would like to recommend biomedical researchers to evaluate the validation criteria of an analytical method prior to its use for determination of MDA levels in biological samples. Full details of such criteria were reported in the guidelines of the Food and Drug Administration (FDA) for biological analysis.^39^ According to our observations, most of the criteria for MDA analysis do not successfully fulfill the FDA requirements. This shortcoming in the method validation criteria could result in non-reliable MDA levels found in different research papers even measured by a single analytical method and consequent controversial discussion on the clinical findings.

In conclusion, although saliva sampling, processing and analysis are simpler than well-established blood sampling due to its simpler matrix, one should consider some restrictions of saliva sampling. The analyte concentration in saliva could be affected by stimulated or non-stimulated sampling procedure, the amount of water intake, and also intake of some drugs. On the other hand, simpler matrix of saliva in comparison with plasma or serum provides more advantages from analytical point of view. In addition, the very wide range of MDA concentrations in saliva is questionable and should be re-investigated. Concerning the above-mentioned points researchers should consider analytical validation criteria to evaluate the reliability of the obtained results on salivary concentrations of MDA and other biomarkers under investigation. There is no doubt on the role of oxidative stress in the etiology of many oral or systemic diseases, but we strongly believe that MDA is not a reliable biomarker for oxidative stress not only in saliva but also in serum/plasma samples.

## Competing interests

The authors declare no competing interests with regards to authorship and/or publication of this article.
